# Volatiles of the Predator *Xylocoris flavipes* Recognized by Its Prey *Tribolium castaneum* (Herbst) and *Oryzaephilus surinamensis* (Linne) as Escape Signals

**DOI:** 10.3390/insects16010031

**Published:** 2024-12-31

**Authors:** Shaohua Lu, Li Yang, Zonglin Wu, Mingshun Chen, Yujie Lu

**Affiliations:** 1 School of Food and Strategic Reserves, Henan University of Technology, Zhengzhou 450001, China; shaohualu08@163.com (S.L.); yangli139039@163.com (L.Y.); wzl19960710@163.com (Z.W.); 2 Department of Entomology, Kansas State University, Manhattan, KS 66506, USA; mchen@ksu.edu; 3 School of Grain Science and Technology, Jiangsu University of Science and Technology, Zhenjiang 212100, China

**Keywords:** natural enemy, olfactory, volatile, stored product pests, repellents

## Abstract

The olfactory sensory system plays vital roles in daily activities, such as locating mate partners, foraging, and risk avoidance. Natural enemies can locate their prey through characteristic volatiles. However, little is known about whether prey can recognize the volatiles of their predators and if this recognition can increase the efficiency of prey escaping from predators. *Xylocoris flavipes* is a predator of *Tribolium castaneum* (Herbst) and *Oryzaephilus surinamensis* (Linne) that has been widely used in stored pest control. Herein, we analyze the volatile components of *Xylocoris flavipes* and their impacts on the olfactory behavior of *T. castaneum* and *O. surinamensis*. We found that *T. castaneum* and *O. surinamensis* preferred blank air rather than odors of *X. flavipes* and *X. flavipes* emissions, which significantly decreased the orientation preference of *T. castaneum* and *O. surinamensis* to wheat. *X. flavipes* emits three major volatiles, including linalool, α-terpineol, and geraniol. Y-tube bioassays showed that *T. castaneum* and *O. surinamensis* can recognize linalool and geraniol at certain concentrations, especially at 200 μg/mL. EAG recordings verified that linalool and geraniol elicit higher olfactory responses in the two pests, but very small EAG responses were observed in the insects to α-terpineol. A further repellency evaluation also proved that linalool and geraniol are repellent to the two pests, and this repellency can be slightly enhanced by mixing them together. *T. castaneum* and *O. surinamensis* can recognize the predator *X. flavipes* by perceiving its volatiles and using them as signals for escaping. The two most potent volatiles, linalool and geraniol, may have potential values as repellents in controlling pests in these two stored products.

## Simple Summary

The sense of smell helps insects perform essential behaviors like finding mates and food and avoiding dangers. Predators use unique smells to locate prey, but it is less understood if prey can recognize these predator odors and use them to escape. In this study, we examined how *Tribolium castaneum* (Coleoptera: Tenebrionidae) and *Oryzaephilus surinamensis* (Coleoptera: Silvanidae), two common pests, react to the predator *Xylocoris flavipes*, widely used in pest control. We found that these pests avoid *X. flavipes* odors, particularly the volatiles, linalool and geraniol, which decrease their attraction to food sources. These compounds show strong potential as natural repellents for pest management.

## 1. Introduction

*Tribolium castaneum* (Herbst) (Coleoptera: Tenebrionidae) and *Oryzaephilus surinamensis* (Linne) (Coleoptera: Silvanidae) are two major stored product pests that cause heavy economic losses to stored grains, including wheat, rice, barley, corn and oats, and processed food products such as flour, pasta, cereals, biscuits and spices. These losses are equivalent to the grain output from millions of hectares of grain fields [1,2]. Traditionally, chemical insecticides such as phosphine fumigation have been extensively used in managing these two pests [3]. However, the overuse of these chemicals has directly resulted in the development of resistance in insects to phosphine [4,5]. Insect resistance results in increased doses of insecticides for subsequent application, which magnifies the side effects of insecticides, including environmental contamination and potential long-term insecticide residues in processed food [6,7]. High levels of resistance in *T. castaneum* and *O. surinamensis* to phosphine have been documented in several recent publications [5,8,9]. Innovative pest control strategies are urgently needed, especially for stored product insects, since they will be directly processed for human consumption.

Volatile-mediated communications can occur between conspecific or hetero-specific living organisms, such as between insects and plants and between insects and their natural enemies [10,11]. Infochemicals have been used in pest control either by incorporating them into insect lures or being used as repellents, depending on the nature of the volatiles and their interactions with other species [12,13,14]. Sex pheromones and volatiles emitted from host plants are especially useful and have been studied extensively [15,16]. For example, sex pheromones from several Lepidopteran and Coleopteran species have been studied in great detail and are widely used for pest control in agricultural settings [17,18,19]. Volatiles emitted from host plants are attractive to natural enemies when they are attacked by herbivore insects. For example, plants attacked by the cotton aphid *Aphis gossypii* release volatiles to attract natural enemies of *Hippodamia variegata* [20]. Volatile organic compounds are critical cues that enable prey to perceive danger by recognizing the presence of predators through these chemical signals [21]. Preys often respond to predators by modifying their behavior to reduce risk [22]. For example, the adult Colorado potato beetle *Leptinotarsa decemlineata* Say is resistant to predation by the spined soldier bug *Podisus maculiventris* Say, yet they still display behavioral changes, such as reduced feeding, when these predators are present [23]. Similarly, when the billbug *Sphenophorus parvulus* Gyllenhal detects predator odors, it exhibits avoidance behavior, indicating that these volatile signals may drive their observed behavioral changes [24]. However, it remains to be studied whether insects can recognize the volatiles emitted from their natural enemy as an escape strategy.

Both the larvae and adults of *Plutella xylostella* (Lepidoptera: Plutellidae) exhibit a behavior that allows them to migrate away from the volatile heptanal [25]. Interestingly, heptanal is one of the volatiles emitted by *Cotesia vestalis*, a predator of *P. xylostella*, implying that insects may recognize the risk through the body volatiles of their natural enemies. *Xylocoris flavipes* (Hemiptera: Anthocoridae) is one of the natural enemies used in controlling stored product pests, such as *Ephestia cautella* (Walker), *T. castaneum*, *Plodia interpunctella* (Hübner), *Corcyra cephalonica* (Stainton), *O. surinamensis*, and *Tribolium confusum* (Du Val) [26]. Here, we use the two stored product pests, *T. castaneum* and *O. surinamensis*, as well as their natural enemy, *X. flavipes*, as a research model to explore this issue. Firstly, we investigated the effects of *X. flavipes* emissions on the olfactory behaviors of *T. castaneum* and *O. surinamensis* in response to food resources. Subsequently, the volatiles emitted by *X. flavipes* were analyzed, and the impacts of specific volatile or volatile combinations on the migration behavior of *T. castaneum* and *O. surinamensis* were examined. A comprehensive understanding of interactions among insect pests and their natural enemies could lead to improving the application of natural enemies in pest management.

## 2. Materials and Methods

### 2.1. Insects

The *X. flavipes* strain was obtained from the Guangzhou Institute of Grain Science (Guangdong, China), which has been reared for many years in the insect laboratory of Henan University of Technology (Zhengzhou, China) using *Plodia interpunctella* larvae in a glass jar (diameter: 10 cm, high: 12 cm). The insect-rearing bottles were placed into an incubator set at a temperature of 30 ± 1° C and a relative humidity of 70 ± 5% (light:dark = 0:24). *T. castaneum* and *O. surinamensis* were reared with wheat flour following a standard procedure [27].

### 2.2. Odor Choice Selection Bioassay

The choice assay followed the methodology described by Lu et al. [27]. Briefly, the setup included three different odor sources: (1) five pairs of *X. flavipes* adults, (2) 5 g of wheat, and (3) 5 g of wheat containing five pairs of *X. flavipes*. Clean air served as the control. Odor sources were placed in separate bottles (height: 12 cm; diameter: 5 cm), which were then connected to a Y-tube (base tube length: 10 cm; internal diameter: 2 cm, arms angle: 60°). The flow of the odor was blown into the arm tubes at 0.3 L/min. After 20 min, the test insects were individually released at the base of the Y-tube, and their behavior was recorded. When the insect crossed 1/3 of an arm within 5 min, it was regarded as a choice [28]. The bioassay was conducted under dark conditions. More than 65 pairs of *T. castaneum* and *O. surinamensis* adult insects were tested, respectively. Ten insects were used in each test of the choice experiment. The positions of different odor sources were exchanged after every two tests.

### 2.3. Volatile Analyses of X. flavipes

Volatiles were collected from *X. flavipes* by transferring five pairs of adults to an empty 50 mL jar. A solid phase microextraction head (50/30 μm DVB/CAR/PDMS coating, Supelco, St. Louis, MO, USA) was used to adsorb the volatiles. The extraction head was inserted into the jar and left at 30 °C for 30 min. After adsorption, the head was removed and directly inserted into the GC-MS (Model: QP2010 ultra; Shimadzu, Japan) for analysis.

The volatile components of *X. flavipes* were identified using a Shimadzu 2010 GC-MS (Shimadzu, Japan) equipped with a DB-5MS column (30 m × 0.25 mm × 2.5 μm). The chromatographic conditions were as follows: split injection; sample inlet temperature at 250 °C; and column temperature from 45 °C to 250 °C at a 5 °C increase per minute and then at a constant temperature of 250 °C for 30 min. MS conditions included an interface temperature of 250 °C, ion source temperature of 230 °C, ionization mode EI, electron energy of 70 eV, and scanning mass range of 50–500 amu. Each group of experiments was repeated five times [29]. The characterization of volatile compounds was performed by comparing the mass spectra with the data system library (National Institute of Standards and Technology, NIST 14.0) and authentic standards [27]. The retention index was calculated based on Vandendool and Kratz [30].

### 2.4. Olfactory Preference of Pests for Chemicals

The tested chemicals were diluted with paraffin oil (Aladdin, Shanghai, China) to prepare the following concentrations: 10, 100, 200, 300, 132, 400, 500, and 1000 μg/mL. In total, 200 µL of these solutions were deployed on a filter paper strip (2.5 cm × 1 cm) and placed into a glass bottle, which was then connected to an arm of the Y-tube. Paraffin oil was utilized as the control. Twenty insects were tested individually and set as one replication. Three replications were carried out for each test [31].

### 2.5. Electroantennogram Recordings of T. castaneum and O. surinamensis to Chemicals

Electroantennogram (EAG) recordings were used to evaluate the antennal responses of pests to individual chemicals. Solutions with the highest observed repellency were selected as the odor stimuli. Specifically, 200 μg/mL of linalool (Aladdin, Shanghai, China) and geraniol (Aladdin, Shanghai, China), and 300 μg/mL of α-terpineol (Aladdin, Shanghai, China) were used for *T. castaneum*, while 400 μg/mL of α-terpineol was used for *O. surinamensis*. First, the antenna of 3-day-old adults was excised from the base and mounted between glass electrodes filled with saline (Takara, Dalian, China) [32]. The electrical conductivity between the antennal preparation and an IDAC-2 amplifier (Syntech Laboratories, Hilversum, The Netherlands), connected to a PC equipped with the Software EAG Pro Version 1.1 (Syntech Laboratories, Hilversum, The Netherlands), was maintained using AgCl-coated silver wires.

Prior to the EAG experiments, 20 μL of the test solution was applied to a filter paper strip (2.5 cm × 1 cm), which was then placed inside a Pasteur pipette (15 cm length) to serve as an odor cartridge. Once a stable baseline was established, the antenna was stimulated for 1 s with an airflow of 150 mL/min, controlled by the air stimulus system. Each antenna was first stimulated with paraffin oil (blank control) and then with a volatile source for testing. Each test was run on 6 different antennae [33,34]. Before the difference analyses were performed, the obtained data were checked for normal distribution.

### 2.6. Repellency Evaluation of Linalool, Geraniol, and Their Mixtures

**Figure 1:**
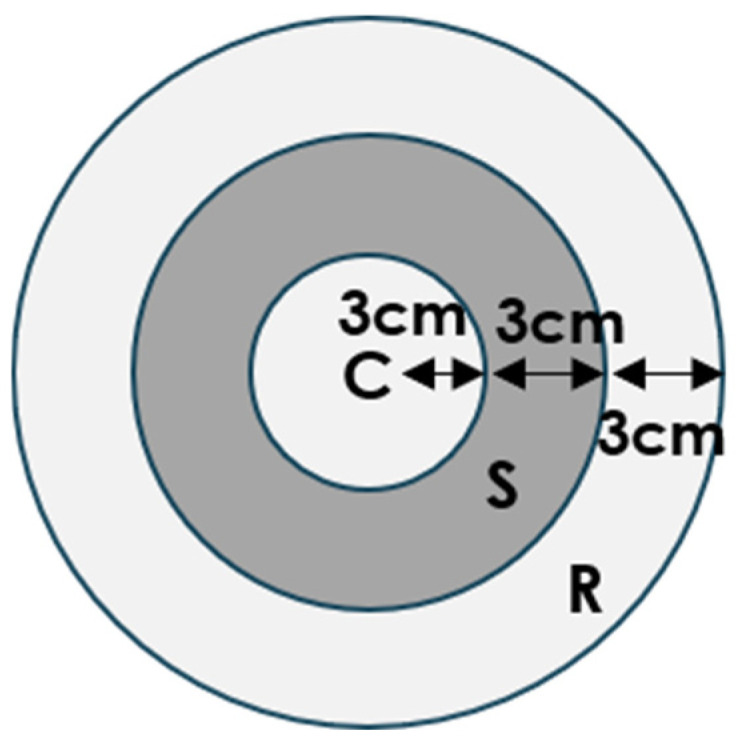
Olfactory assay arena. Wheat was placed in the C region; the annular filter paper was soaked with different solutions and was placed in region S. The test insect was released at the outer edge of the S region.

The bioassay was conducted to evaluate the potential application of two chemicals for the control of the two stored pests. Briefly, 3 g of wheat was placed at the C region inside a Petri dish (diameter = 18 cm) (Figure 1). An annular filter paper was soaked with 200 μg/mL of linalool, geraniol, and mixtures containing 200 μg/mL of both compounds. The filter paper soaked with paraffin oil was placed in the S region and considered the control. Subsequently, adult insects were released at the outer edge of the S region individually. After 5 min, the location of the released insects was recorded. A total of 120 *T. castaneum* and *O. surinamensis* adults were tested in each group, respectively. The repellency index (RpI) was calculated with Equation (1) as follows:

RpI = (C − R)/C(1)

C: number of insects in the C region. R: number of insects in the R region.

### 2.7. Data Analyses

For the choice assay and Y-tube test, the number of insects in each group was compared using the Chi-square test (*p* < 0.05). EAG amplitudes were analyzed using a one-way ANOVA (*p* < 0.05, Duncan’s test). All analyses were conducted using SPSS (Statistical Package for the Social Sciences) software, version 26.0 (IBM).

## 3. Results

### 3.1. Odor Choice Selection Bioassay

**Figure 2:**
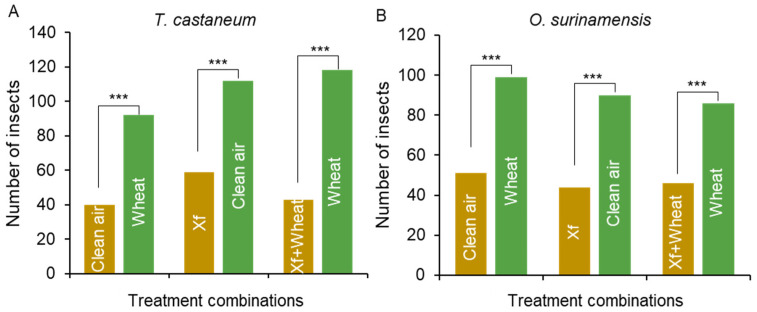
Y-olfactometer tests for pests on two stored products. (**A**): Preference of *T. castaneum* towards different odor sources. (**B**): Preference of *O. surinamensis* towards different odor sources. Xf: *X. flavipes*. “***” means *p* < 0.001. The Chi-square test was used to calculate the difference between each comparison.

In the olfactory bioassay, both *T. castaneum* and *O. surinamensis* showed a preference for migration towards the odor of wheat, compared with the clean air control (*p* < 0.001). However, this selection preference decreased with the presence of natural enemy *X. flavipes* in the wheat. This observation implies that *T. castaneum* and *O. surinamensis* can recognize volatiles released by *X. flavipes* and use these volatiles as warning signals for the presence of dangerous predators (Figure 2A,B).

### 3.2. Volatile Analyses of X. flavipes

**Figure 3:**
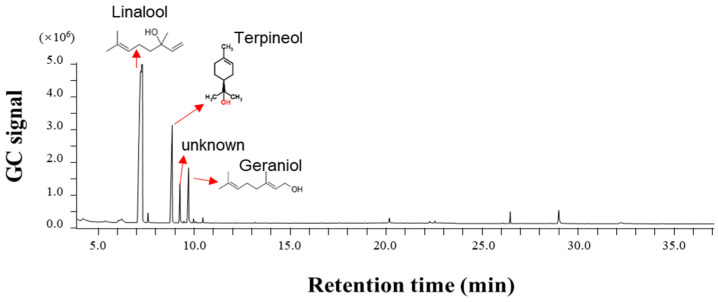
Volatile profile analyses of *Xylocoris flavipes*. The GC signal showed a relative abundance of chemicals in volatile profiles.

Based on the olfactory bioassays of *T. castaneum* and *O. surinamensis* towards different odor sources, we collected and analyzed the volatile profile of *X. flavipes*. Three dominant chemicals were identified, including linalool, α-terpineol, and geraniol. The linalool showed the highest percentage among the three volatiles (Figure 3, Table 1).

### 3.3. Olfactory Preference of Pests for Chemicals

**Figure 4:**
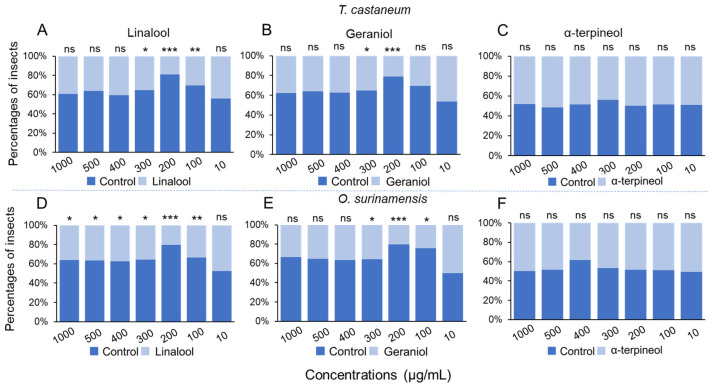
Olfactory bioassay assessing the response of *Tribolium castaneum* and *Oryzaephilus surinamensis* to *Xylocoris flavipes* volatiles. (**A**–**C**) The migration response of *T. castaneum* towards linalool (**A**), geraniol (**B**), and α-terpineol (**C**). (**D**–**F**) The migration response of *O. surinamensis* towards linalool (**D**), geraniol (**E**), and α-terpineol (**F**). “ns” means *p* > 0.05; “*” means *p* < 0.05; “**” means *p* < 0.01; and “***” means *p* < 0.001. The Chi-square test was used to calculate the difference between each comparison.

Y-tube bioassays were conducted to evaluate the olfactory effects of the three volatiles from *X. flavipes* on the migration behavior of *T. castaneum* and *O. surinamensis*. Linalool and geraniol showed a repellent impact on both *T. castaneum* and *O. surinamensis* at some tested concentrations (Figure 4). Specifically, the most effective concentration of linalool and geraniol was 200 μg/mL (Figure 4A,B,D,E). However, at these concentrations, α-terpineol did not exhibit any repellent effect on any insect (Figure 4C,F).

### 3.4. Electroantennographic Responses of T. castaneum and O. surinamensis to Xylocoris flavipes Volatiles

**Figure 5:**
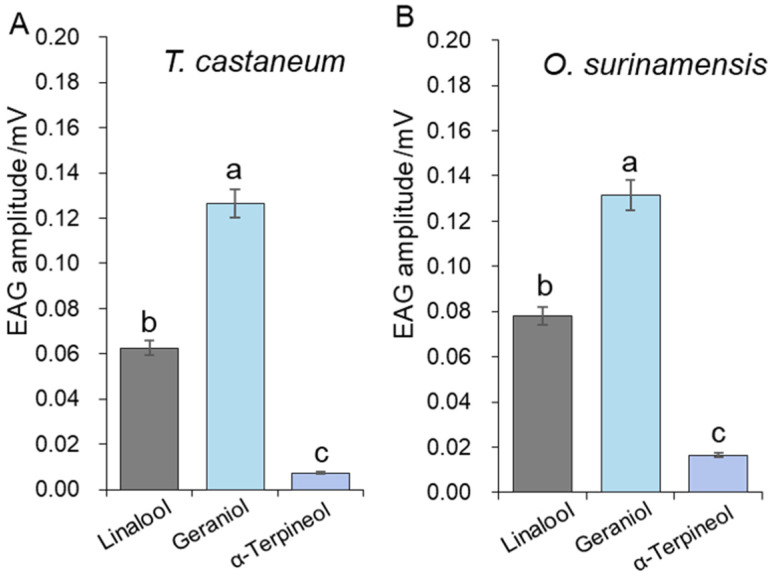
EAG responses of *Tribolium castaneum* and *Oryzaephilus surinamensis* to different volatiles. (**A**): The preference of *T. castaneum* towards different volatiles. (**B**): The preference of *O. surinamensis* towards different volatiles. The data are presented as the mean ± standard error. EAG values in the graph are given minus the blank response as the control. Different letters indicate significant differences. One-way ANOVA was used to calculate the statistical difference among groups (*p* < 0.05).

To evaluate the olfactory responses of *T. castaneum* and *O. surinamensis* to *Xylocoris flavipes* volatiles, the electroantennogram (EAG) responses to these chemicals were recorded (Figure 5). Both insects showed significant EAG responses to geraniol and linalool (Figure 5) but were less sensitive to α-terpineol, which is reflected at the lowest EAG amplitude (about 0.008 mV).

### 3.5. Repellency Evaluation of Linalool, Geraniol, and Their Mixtures

**Figure 6:**
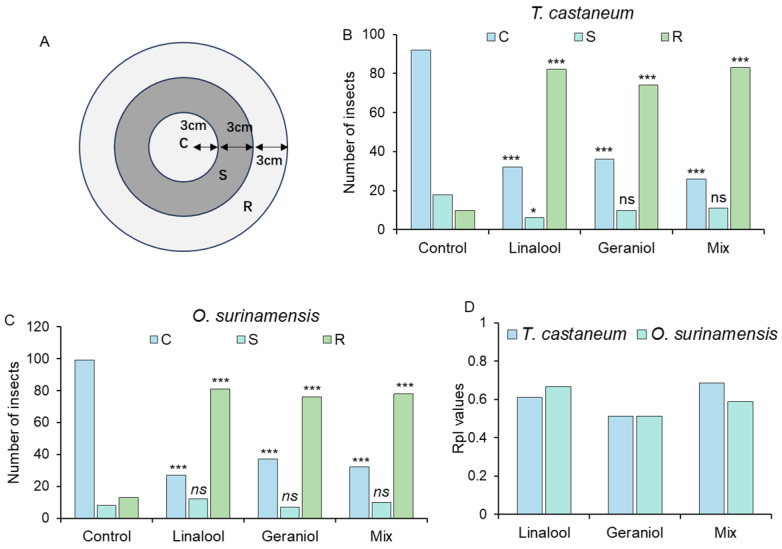
Repellent effect of geraniol and linalool on *Tribolium castaneum* and *Oryzaephilus surinamensis*. The sketch map of the bioassay is shown in (**A**); the number of *T. castaneum* is shown in (**B**); and that of *O. surinamensis* in (**C**) is shown under the exposure of different solutions. RpI values of solutions on *T. castaneum* and *O. surinamensis* are displayed in (**D**). “*” = *p* < 0.05; “***” = *p* < 0.001; “ns” = not significant. The Chi-square test was used to calculate the difference between each comparison. The mixed solution contained two solutions of 200 μg/mL of linalool and geraniol. The number of insects in each region exposed to different chemicals was compared with that in the control.

A bioassay was conducted to examine the potential application of the two chemicals. When paraffin oil was added to the S region (Figure 6A), most of the released insects in the control group migrated from the outer edge of the S region towards the wheat in the C region. In contrast, when the paraffin oil was replaced with 200 μg/mL of linalool or geraniol, most of the released insects moved toward the R region (Figure 6B,C). This result implied that the attractiveness of wheat was diminished by the presence of the two volatiles. When the two volatiles were mixed at a 1:1 ratio, we did not observe a significant increase in the repellency effect (Figure 6D).

## 4. Discussion

In this study, we uncovered an interesting interaction between prey and their predators. *T. castaneum* and *O. surinamensis* can perceive volatile chemicals, such as linalool, α-terpineol, and geraniol, from their natural enemy *X. flavipes*. Two of the volatiles, linalool, and geraniol, were repellents to the insects, which may be useful for the design of repellency strategies to control these pests.

Chemical signal-mediated avoidance of natural enemies by prey has been reported in many cases. For example, the spotted cucumber beetle *Diabrotica undecimpunctata* reduces feeding when exposed to leaves that contain semiochemicals of the wolf spider *Hogna helluo* [35]. Gravid females of the mosquitoes *Culiseta longiareolata* and *Anopheles gambiae* are repelled from oviposition sites by two semiochemicals released by the predatory backswimmer *Notonecta maculate* [36]. When aphids are attacked by predators, they release alarm pheromones, such as (E)-β-farnesene, to warn nearby conspecifics to flee. These pheromones not only induce escape behaviors in other aphids but also attract predatory insects to the location [37]. Here, we observed that when the predator *X. flavipes* is present along with wheat, the number of *T. castaneum* and *O. surinamensis* migrating towards wheat decreases (Figure 2). We speculated that there were repellent cues in the volatiles released by the predator *X. flavipes*. Diverse functions of insect volatiles have been mostly focused on the attraction between mating partners and repulsion between con- or hetero-species [38]. Very limited studies have been carried out to reveal the communication roles of volatiles in prey to detect the presence of their natural enemies. Previous studies indicated that exposure to a natural enemy results in an adverse effect on the prey’s growth and population development, implying that detectable cues from natural enemies can be perceived by prey [39,40].

Our Y-tube bioassay results showed that linalool and geraniol are repellent to *T. castaneum* and *O. surinamensis*. Specifically, linalool and geraniol showed the highest repellency at 200 μg/mL. Although these chemicals were also released by other organisms in nature, this avoidance behavior may be a dose-dependent response of the two pests. That means organisms show differences in sensitivity to various concentrations of volatile chemicals. For example, our previous study results showed that two evolutionarily related weevils, the maize weevil *Sitophilus zeamais* and the rice weevil *Sitophilus oryzae*, show different levels of sensitivity to 2-ethylhexanol, piperitone, and (+)-Δ-cadiene. This sensitivity difference induces them to migrate to preferred food resources [27]. Furthermore, our Y-tube test results also showed that the two stored product pests displayed varied sensitivity to different concentrations of chemicals (Figure 4). Therefore, linalool and geraniol induced avoidance behavior in both pests, possibly in a dose-dependent response to a certain ratio of the volatile blend.

In the EAG test, geraniol and linalool induced greater antennal response than α-terpineol (Figure 5). Previous studies have suggested that some essential oils containing geraniol are repellent to herbivore insects [41]. Pajaro-Castro et al. [42] reported that linalool is repellent to *T. castaneum* with a repellent concentration 50 (RC_50_) value of 0.11 μL/cm^2^, which aligns with the results of our bioassays. The three main chemicals emitted by *X. flavipes* are all monoterpenes, most of which have been proven harmful to the development and metabolism of herbivorous insects and impede their feeding behavior [43,44,45]. Also, when adding solutions of 200 μg/mL of geraniol and linalool as well as their mixture, along with wheat, the orientation preference of the two pests to wheat significantly decreased (Figure 6B,C). This result not only confirms the repellency of the two chemicals but also suggests that they could serve as escape signals for pests. The olfactory-evoked responses rely on the perception of chemical signals in the environment. In this process, chemical signals were bound and transported by odorant-binding proteins (OBPs), chemosensory proteins (CSPs), and olfactory receptors (ORs) to olfactory receptor neurons (ORNs), resulting in various behavioral changes [46,47]. Further studies are needed on the specific sensory proteins involved in predator recognition by both pests. Meanwhile, the application of the two repellent chemicals in a storage environment should be further studied. Specifically, more combined mixtures should be explored and evaluated in practical conditions.

## 5. Conclusions

*T. castaneum* and *O. surinamensis* can recognize the predator *X. flavipes* by detecting its volatiles, particularly linalool and geraniol, which serve as escape signals. These two volatiles show significant repellency effects on the pests, especially at the concentration of 200 μg/mL. Thus, linalool and geraniol appear to have potential as effective repellents in controlling these stored product pests.

## Figures and Tables

**Table 1:** Major components in the volatile profile of *X. flavipes*.

No.	Retention Time/Min	Retention Index	Compound	Relative Content/%
1	6.45	1082	linalool	67.55 ± 7.33
2	8.28	1079	α-terpineol	14.86 ± 4.37
3	8.72	1125	geraniol	10.43 ± 3.62

## Data Availability

All data generated or analyzed during this study are included in this published article.
